# Effects of Dermatan Sulfate from Marine Invertebrate *Styela plicata* in the Wound Healing Pathway: A Natural Resource Applied to Regenerative Therapy

**DOI:** 10.3390/md20110676

**Published:** 2022-10-28

**Authors:** Vanessa S. Rizzo-Valente, Maria A. Fusco, Renata M. M. L. Cruz, Rachel A. Santos, Lucas S. Silva, Roberta C. Escaleira, Daniel F. Schulz, Shana P. C. Barroso, Bruno L. Miranda, Daniela Z. Santos, Marcelo L. Gregório, Rodrigo J. A. Guerra, Mauro S. G. Pavão

**Affiliations:** 1 Biomedical Research Institute, Marcílio Dias Naval Hospital, Brazilian Navy, Rio de Janeiro 20725-090, Brazil; 2 Laboratory of Biochemistry and Cell Biology of Glycoconjugates, Clementino Fraga Filho University Hospital and Institute of Medical Biochemistry Leopoldo De Meis, Federal University of Rio de Janeiro, Rio de Janeiro 21941-913, Brazil

**Keywords:** ascidian, dermatan sulfate, fibroblast, *Styela plicata*, wound healing

## Abstract

Acute and chronic dermatological injuries need rapid tissue repair due to the susceptibility to infections. To effectively promote cutaneous wound recovery, it is essential to develop safe, low-cost, and affordable regenerative tools. Therefore, we aimed to identify the biological mechanisms involved in the wound healing properties of the glycosaminoglycan dermatan sulfate (DS), obtained from ascidian *Styela plicata*, a marine invertebrate, which in preliminary work from our group showed no toxicity and promoted a remarkable fibroblast proliferation and migration. In this study, 2,4-DS (50 µg/mL)-treated and control groups had the relative gene expression of 84 genes participating in the healing pathway evaluated. The results showed that 57% of the genes were overexpressed during treatment, 16% were underexpressed, and 9.52% were not detected. In silico analysis of metabolic interactions exhibited overexpression of genes related to: extracellular matrix organization, hemostasis, secretion of inflammatory mediators, and regulation of insulin-like growth factor transport and uptake. Furthermore, in C57BL/6 mice subjected to experimental wounds treated with 0.25% 2,4-DS, the histological parameters demonstrated a great capacity for vascular recovery. Additionally, this study confirmed that DS is a potent inducer of wound-healing cellular pathways and a promoter of neovascularization, being a natural ally in the tissue regeneration strategy.

## 1. Introduction

**Figure 1:**
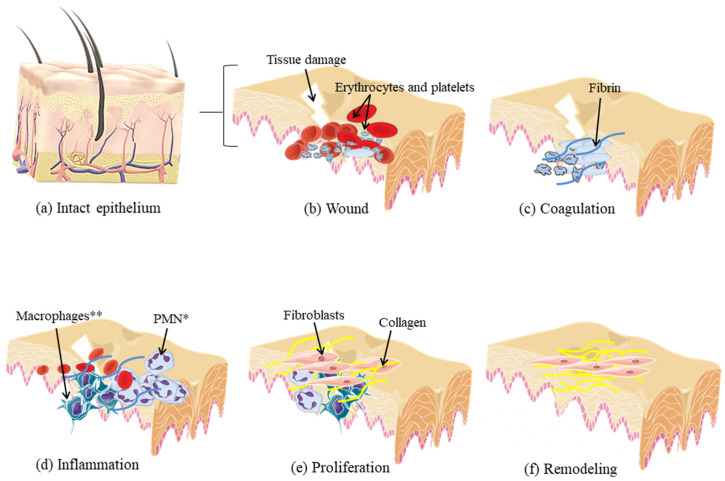
Biological stages of wound healing. (**a**–**d**) After the wound, the inflammatory stage occurs, including the leakage of blood components (followed by platelet aggregation, blood clotting, and inflammatory process); (**e**) a proliferative stage of keratinocytes, fibroblasts, and endothelial cells; (**f**) and a final stage of granular tissue remodeling and re-epithelialization. In normal healing activity, fibroblasts act from the end of the inflammatory phase (24–48 h after the injury) until the completion of tissue re-epithelization. For that, migration occurs to the wound site, where fibroblasts infiltrate and proliferate [3]. Then, fibrin degradation occurs through the production of matrix metalloproteinases (MMPs), and substitution by extracellular matrix (ECM) components, such as collagen I-IV, XVIII, glycoproteins, proteoglycans, laminin, thrombospondin, and glycosaminoglycans (GAGs) [4]. This newly constituted matrix provides support and regulates fibroblast migration and activity, as well as produces signaling molecules for the onset of angiogenesis, formation of granulation tissue, and re-epithelialization [5].

Skin lesions are challenging morbidity concerns in clinical practices. Biological events following the injury result in tissue healing, which is a progressive and dynamic process, composed of three highly integrated and overlapping stages: an inflammatory stage consisting of the leakage of blood components, with platelet aggregation, blood clotting, and migration of inflammatory cells; a proliferative stage, involving the migration and proliferation of keratinocytes, fibroblasts, and endothelial cells, resulting in re-epithelialization and formation of a granular tissue; and a final stage of tissue remodeling, restoring the skin integrity and its function [1,2] as shown in Figure 1. 

Molecular and cellular mechanisms that regulate the wound repair process have not yet been fully elucidated; without this knowledge, the progress of current therapies can be inefficient [6]. Fortunately, several cytokines and molecules that act synergistically to complete the tissue repair process have been identified. In recent studies, the role of transforming growth factor-β (TGF-β) and components of the fibroblast growth factor (FGF) family [7,8,9] have been well elucidated. TGF-β is a multifunctional growth factor, which has pleiotropic effects on healing through the regulation of cell proliferation, differentiation, production of ECM components, and immunomodulation [10]. The fibroblast growth factors FGF-2 and FGF-7 participate in the main stages of inflammation, repair, and regeneration. FGF-2 signaling is related to the proliferation of fibroblasts and endothelial cells, as well as cell migration epithelial cells. FGF-7, or keratinocyte factor, contributes to epithelial cell regeneration [11]. However, the participation of other growth factors and cytokines in the modulation of extracellular matrix components during the healing process still needs clarification.

The structural and functional diversity of proteoglycans is evident, which is why they are present in the ECM anchored to different types of proteins, with a variable number of GAGs chains, and on the surfaces of many cells [12]. Some molecules present in ECM, such as GAGs, play essential roles in wound repair throughout the healing process. Dermatan sulfate (DS) occurs in a variety of organisms and is the main GAG in the skin and connective tissues of vertebrates, representing about 36–78% of the total sulfated polysaccharides in wound fluids [13]. In general, proteoglycans containing DS are essential to cell growth (as modulators of growth factors and collagen functions), tissue organization, and the maturation of specialized tissues, as well as in the skin’s tensile strength [14]. 

After a skin injury, binding of DS to FGF-7 occurs, which enhances the growth factor activity. As a result, there is an increase in the proliferation of keratinocytes, through a mechanism that involves the phosphorylation of mitogen-activated protein kinases (MAP kinases), mediated by the binding of FGF 7 to the FGFR2 IIIb receptor [8]. Similarly, DS stimulates the proliferation of fibroblasts mediated by FGF-2 [9]. The interaction of DS with TGF-β modulates its fibrogenic activity, which is important in controlling healing [15].

**Figure 2:**
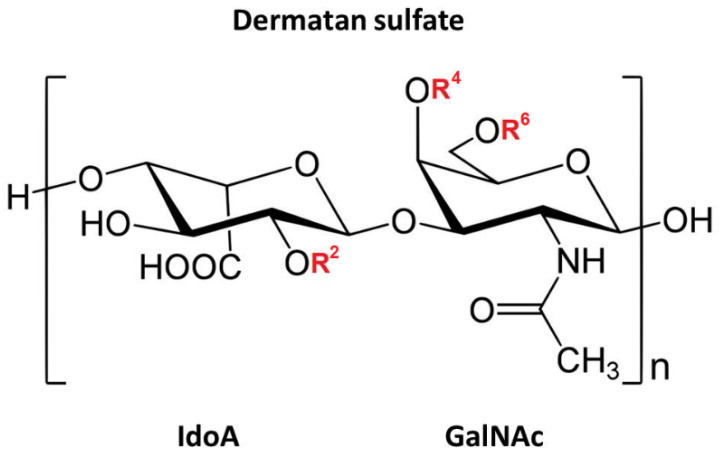
Chemical structure of a disaccharide unit of dermatan sulfate. Highlighted, the polymer section demonstrates typical structural characteristics of dermatan sulfate (DS); however, the repetitive sequence of variously substituted disaccharide units was arbitrarily selected. In red, R^2^, R^4^, and R^6^ correspond to the possibilities of insertion of sulfate residues in positions 2 of iduronic acid (IdoA) and/or 4 and/or 6 in *N*-acetyl galactosamine (GalNAc), respectively, the “n” on the outside refers to the number of times a disaccharide unit can be repeated in a polysaccharide that contains DS.

**Figure 3:**
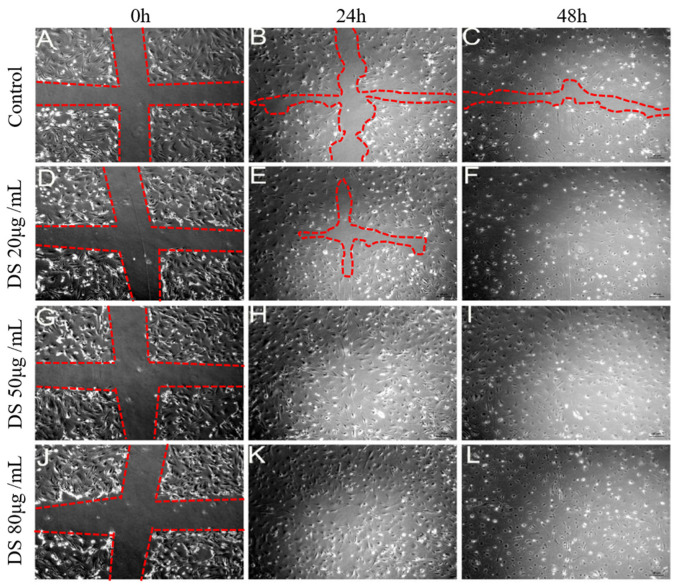
Effect of dermatan sulfate from *Styela plicata* on cell migration. The in vitro scratch assay demonstrated mechanically decellularized areas of murine fibroblast cultures that were evaluated after 24 and 48 h of treatment with 2,4-DS at concentrations of 20, 50, and 80 μg/mL. Cell migration was followed up to 48 h after the monolayer scratching and, under control conditions, areas not occupied by fibroblasts (**B**,**C**) were also observed. In the presence of the DS 20 μg/mL, a gradual reduction in the decellularized area (**E**,**F**) was observed, as in the control. However, using 2,4-DS at concentrations of 50 and 80 μg/mL, it was possible to observe the total occupation of the area in 24 h after treatment (**H**,**K**) and greater dispersion after 48 h (**G**,**L**). DS treatment reduced healing time in vitro. Adapted from [21].

It is known that the DS molecule is constituted by repetitive disaccharide units of *N*-acetylgalactosamine and hexuronic acid (glucuronic and/or iduronic acid), linked by glycosidic bonds of type (1,3) and (1,4), respectively. Hexosamine can contain sulfation at carbon 4 and/ or 6, and hexuronic acid may be sulfated at carbon 2, especially when it is iduronic [16], a fact that explains its heterogeneity (Figure 2). In marine invertebrates, several polymers with biological structures and activities similar to vertebrate mammalian GAGs have been identified. Tunicates are the phylogenetic group of marine invertebrates closest to vertebrates [17]. A particular class of tunicates is formed by ascidians, which are sessile organisms in adulthood, rich in sulfated polysaccharides of pharmacological interest. Among them is DS, isolated mainly from the viscera of the animal, similar to those found in vertebrates, but with differences in the degree and position of sulfation. This molecule was previously isolated from the ascidian *Styela plicata* and tested in experimental models of venous and arterial thrombosis, which showed a direct link between anticoagulant and antithrombotic activities [18]. The biological activity of this polymer was related exclusively to the presence of regions enriched in [IdoA(2-OSO_3_)-GalNAc(4-OSO_3_)]n (n ≥ 3) [19] or 2,4-DS. Besides that, the antimetastatic and antiviral effects of DS have also been described [20]. Furthermore, in previous work by our group, it was observed that the 2,4-DS obtained from *S. plicata* did not present toxicity in the concentration range of 10 μg/mL to 1 mg/mL and was able to promote an increment in the proliferation of murine fibroblasts in vitro, increasing their migration in the healing assay (Figure 3) [21].

Considering the constitutive aspects and pharmacological properties described above, allied to the fact that the DS is devoid of the platelet aggregation effect and does not induce bleeding in vivo tests [22], the present study aimed to investigate the effects of DS: on the enhancing the healing process; such as modulator of the recruitment of inflammatory cells and the signaling of growth factors involved in cell migration and proliferation and, finally, in the expression of genes participating in the healing pathway and other related paths. 

## 2. Results

### 2.1. Isolation and Characterization of Dermatan Sulfate from Styela plicata

**Figure 4:**
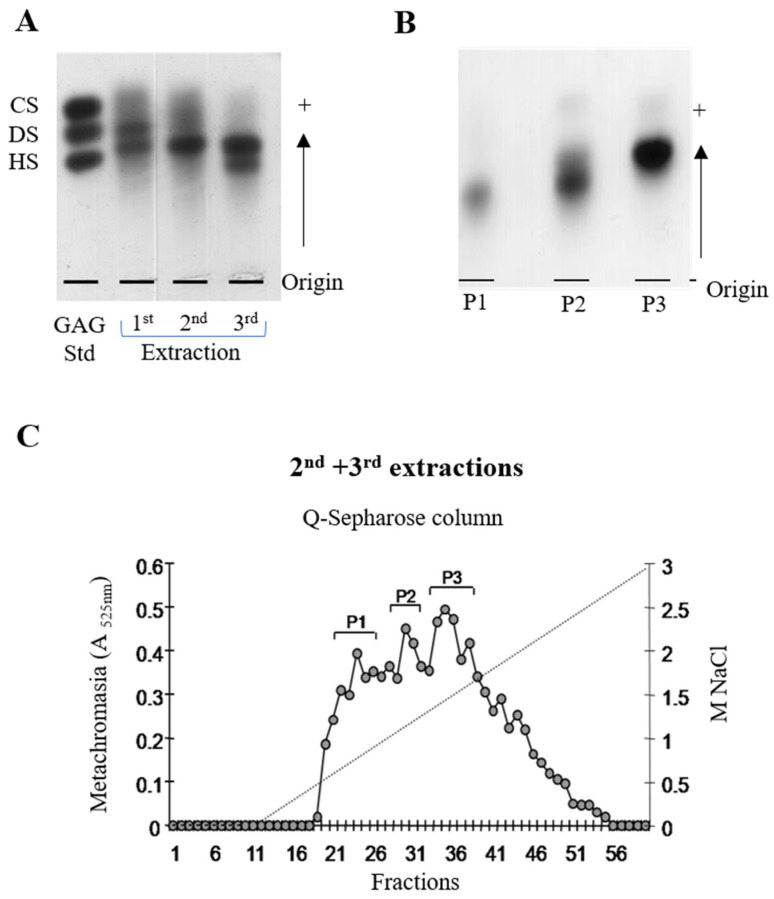
Purification of dermatan sulfate from the viscera of the ascidian *Styela plicata*. (**A**) Agarose gel electrophoresis of the polysaccharides obtained in the first (1st), second (2nd), and (3rd) extractions; (**B**) agarose gel electrophoresis of the polysaccharides obtained from peaks P1, P2, and P3 of the Q-Sepharose column; (**C**) ion-exchange chromatography of the pooled polysaccharides obtained from the 2nd and 3rd extractions on a Q-Sepharose column.

The obtention and isolation of dermatan sulfate from *Styela plicata* ascidian were performed as described in Section 4.2. The disaccharide analysis by the Q-Sepharose column confirmed the DS composition with a high content of 2-*O*-sulfated α-*L*-iduronic acid and 4-*O*-sulfated *N*-acetyl-β-D-galactosamine units (Figure 4), corresponding to more than half of the identified residues, as previously observed and described by Pavão and co-workers [18].

### 2.2. Evaluation of the Effect of Dermatan Sulfate on the In Vivo Healing Model

The macroscopic evaluation of the wounds was performed on days 4, 7, and 17 post-surgery (corresponding to the stages: inflammatory, fibroblastic, and remodeling, respectively, as shown in Figure 1d–f. 

**Figure 5:**
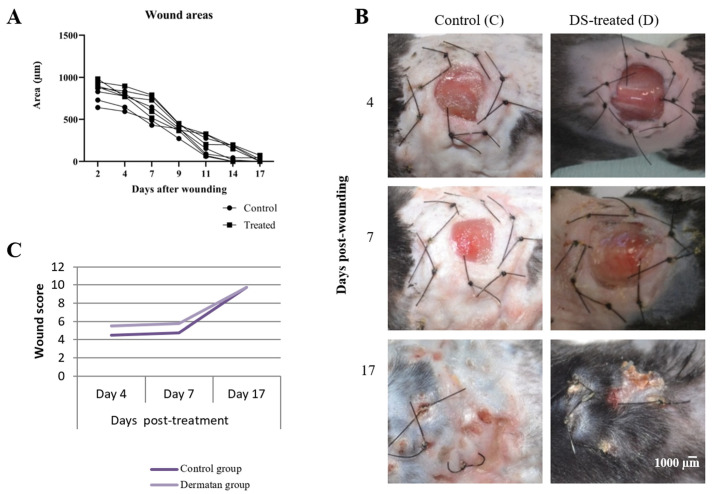
Macroscopic assessment of wound healing progress. (**A**) Quantification of the wound areas. Data are presented as wound area (µm) corresponding to 2, 4, 7, 9, 11, 14, and 17 days post-wounding both in control (NaCl: 0.9%) and 0.25% 2,4-DS-treated mice groups (n = 4 wounds/group). The analysis was performed using two-way ANOVA statistics and do not show significant differences between the analyzed groups. (**B**) Appearance of wound areas. Though both groups presented similar wound areas along the time, the wound closure and the recovery of original characteristics were noticeably accelerated in the skin of 0.25% 2,4-DS-treated mice group, especially from day 17 post-wounding, when considering the presence of complete mice pelage. (**C**) Comparison of the wound score. Ordinal measurement scale: scores ranging from zero (poor healing) to 12 (clinically healed wound). The wound score analysis between both groups showed that at all stages of the healing process, the action of the DS on the wounds was superior to the use of saline (control group).

The quantification of the wound areas from day 0 to 17 post-wounding did not show significant differences between the control and 0.25% 2,4-DS-treated mice groups (Figure 5A and Table S1). Slight bleeding was more noticeable in the animals treated with 2,4-DS during the fibroblastic phase (Figure 5B: D4 and D7) and the wound closure and the recovery of original characteristics were noticeably accelerated in the skin of 0.25% 2,4-DS-treated mice group, especially from day 7 post-wounding, when considering the presence of complete mice pelage. In the same way as the analysis of the wound areas, the comparison of the average score between the groups also showed that at all stages of the healing process, the action of DS on the wounds was similar to the use of saline (control group) (Figure 5C).

**Figure 6:**
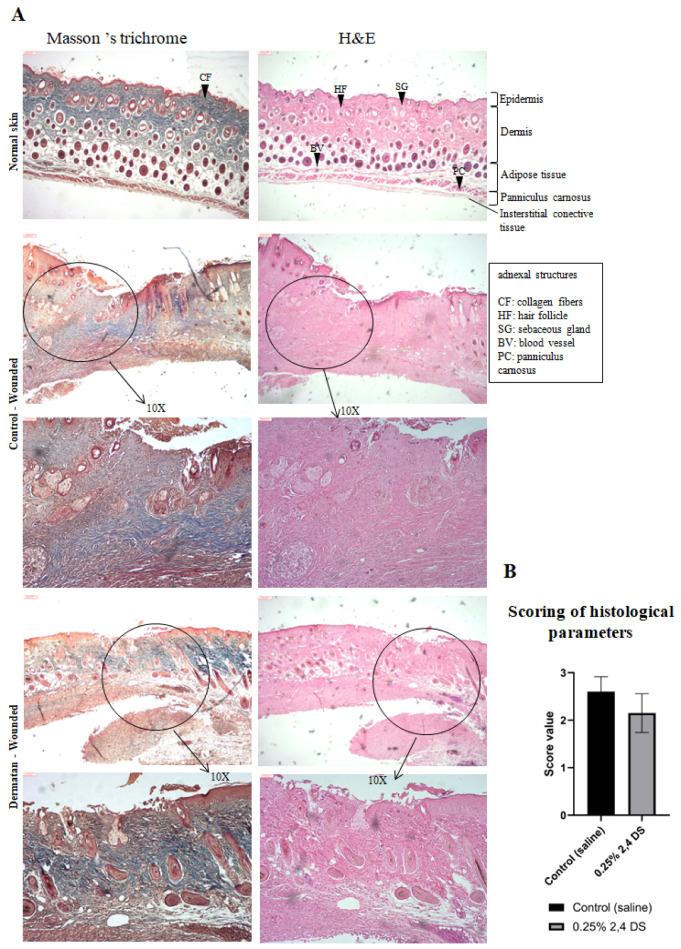
Histological evaluation of wound healing evolution. (**A**) Histological features of wounded tissue. The top images show representative features of normal mice skin under H&E and Masson’s trichrome staining. Satisfactory wound closure is characterized by the formation of all layers related to normal skin, with a large amount of reticular collagen (colored in blue), the presence of new vessels, and total re-epithelization, observed principally on wounded tissue of 0.25% 2,4-DS-treated mice. Images obtained in an optical microscope (X4 and X10 magnification). Scale bar: 6 mm. (**B**) Quantification of the mean histological score. The result of the average score between the five selected parameters classifies the wound as poorly healed (value 3), in the healing process (value 2), and healed/re-epithelized wound (value 1). (*n* = 4 wounds/group) at day 17-post wounding. The analysis was performed using ImageJ^®^ software (Bethesda, MD, USA).

Histological and score analyses were performed based on biopsies on the 17th-day post-surgery, evaluating the parameters: the amount of granulation tissue, collagen pattern, vascular proliferation, and re-epithelization in tissues stained with hematoxylin–eosin and Masson’s trichrome. The microscopic analysis showed dystrophic sebaceous glands and hair follicles in control (saline treatment). In the DS-treated group, histological features of wound healing, such as reticular collagen (in blue) with organized fibers, functional hair follicles, and vascularization were observed (Figure 6A). The results of the parameter scores, based on methodology described in the Table 2, (Section 4.3) resulted in the averages observed in Figure 6B.

### 2.3. Evaluation of Gene Expression by Polymerase Chain Reaction (PCR) Array and Reactome Interaction Analysis

Prior to the gene expression assay, a test run was carried out to assess the quality control of the RNA of each sample of cultured fibroblasts (control and 50 μg/mL DS-treated groups, in biological triplicates), which did not reveal contamination by genomic DNA (Table S2). In addition, the expression levels of two constitutive genes, β-actin and hypoxanthine phosphoribosyltransferase 1 (Actb and Hprt1, respectively), were detected, which demonstrated stable baseline concentration of Hprt1 and non-statistical variation of the Actb gene (Figure S1).

**Figure 7:**
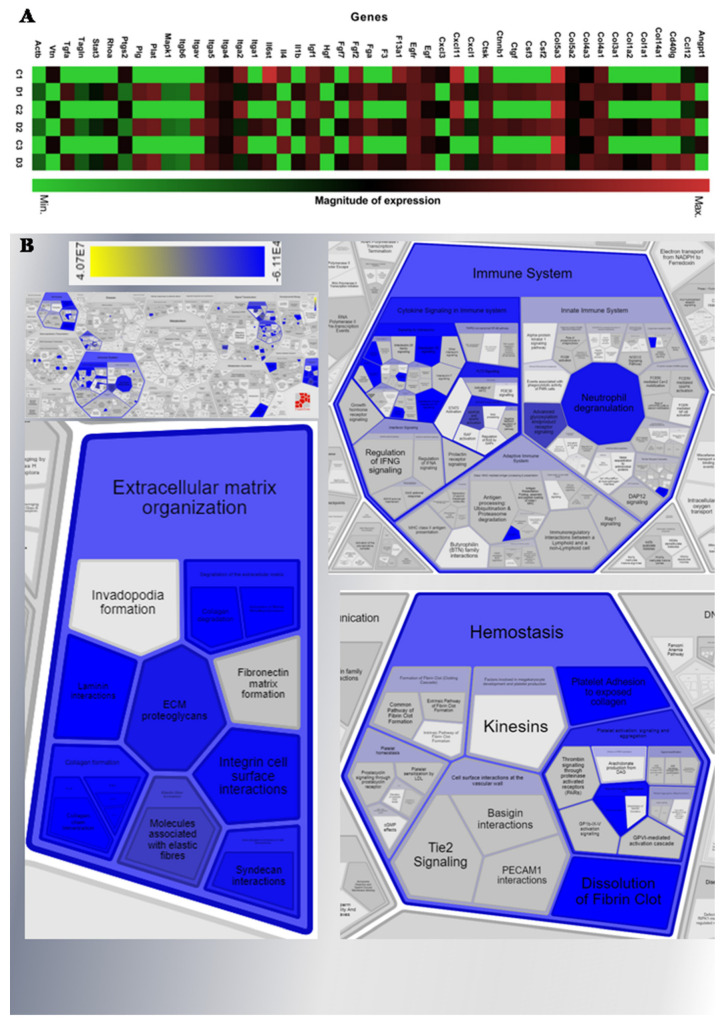
(**A**) Heatmap of gene expression analysis. The in vitro wounded fibroblasts (2,4-DS 50 μg/mL-treated and control groups) were subjected to qPCR analysis to assess the amount of mRNA of 84 genes involved in the wound healing pathway. The expression of target genes was calculated using the ΔΔCT method. (**B**) Relative levels of mRNA by pathway. Reactome shows enrichment analysis of 84 genes from 2,4-DS 50 μg/mL-treated vs. control conditions. Reactome pathway – blue indicates matched entities and the gradient represents significance (*p*-value < 0.05).

As a result of the evaluation of the gene expression profile in the healing pathway, the level of expression of only one (Hsp90ab1) of the five housekeeping genes present in the array (Actb, B2m, Gapdh, Gusb, and Hsp90ab1) was not influenced by the experimental conditions. The Hsp90ab1 gene was selected for normalization, in association with two other genes belonging to the healing pathway, and those also showed basal expression under the conditions tested (Ccl7, also named mcp3, and Egfr). The choice of genes that initially were not part of the endogenous control list, performed automatically by the software, was necessary to replace those that had their expression altered by the treatment. The following outlier genes were excluded from the analysis (CT > 36): Actc1, Cdh1, Col5a1, Ctsl, Itgb3, Tgfb1, Timp1, and Gusb. These genes appeared only in test group arrays. The following genes did not have differential expression detected in either group: Acta2, Il10, Il2, Itga6, Itgb5, Pdgfa, Serpine1, and Tnf. In the treated group, there were 48 genes upregulated and 14 genes downregulated at day 1 post-wounding, as compared to the control group (*p*-value ≤ 0.05) (Figure 7A, detailed in Table S3). 

Through in silico high-performance analysis in the Reactome Pathway Portal (version 3.2) [23], the relationships between the genes modulated in the studied condition and their interactions with the corresponding biological pathways were inferred. These analyses were computationally inferred from an event that was demonstrated in another species through homology mapping (aligning the orthologous identified in the PANTHER database who share at least 75% of identity) [24]. For this evaluation, the list of genes and their respective fold regulation (fr) values (Table S3) were sent as input. The Mmp1a, Tagln, and Tgfbr3 genes were excluded from this analysis because they were not found by the software in the databases consulted.

The biomolecular pathways coordinated by each gene were dismembered (Figure 7B), which revealed the activation of the main route of organization of the ECM, with the induction of collagen formation through the assembly of collagen fibrils and other multimeric structures (gene ontology: 0030198), while the formation of fibronectin matrix and invadopodia were not identified. Regarding the genes related to the organization of the ECM, those responsible for the mechanisms of collagen degradation and activation of matrix metalloproteinases, as well as collagen biosynthesis and modification enzymes (collagen chain trimerization) were activated. In addition, genes responsible for building collagen fibers and other structures, such as the production of molecules associated with elastin fibers, with subsequent fibril crosslinking and anchoring were also activated. Genes involved in the interactions of integrins with the cell surface and of non-membrane integrins with the ECM (such as syndecan and laminin) were also transcribed. Thirty-five genes encoding proteins were identified as part of this mechanism. In addition to the aforementioned pathway, genes that participate in hemostasis were activated in response to platelet aggregation to exposed collagen, with subsequent platelet degranulation and dissolution of fibrin clots. 

The healing event studied also activated the immune system pathway, more specifically the genes that regulate inflammatory processes, such as neutrophil degranulation and secretion of inflammatory cytokines and chemokines. Genes related to cytokine signaling pathways were identified (Fltr subroute, by activating the Raf/Map kinase cascade; regulated by the Map2k and Mapk genes and by activating Mapk3/Erk1 and Mapk1/Erk2). Another activated pathway was cell signaling by second messengers (Pip3 activating signaling by Akt and negative regulation of the Pi3k/Akt network), in addition to Pi5p, Pp2a, and Ier3 regulating Pi3k/Akt signaling. The participation of the chemokine Cxcl11, as well as interleukins (Il4 and Il13; Il6 and Il10), and the signaling by interleukin Shc receptors was also modulated.

In protein metabolism, it was found that the pathway for regulating the transport and uptake of Igf (insulin-like growth factor) was activated by insulin-like growth factor binding protein(s) (Igfbp). 

Several signaling cascades were partially activated: Esr, Wnt, Rho GTPases, tyrosine receptor kinases (Egfr, Met, Fgfr1 and 2, Vegf, Pdgf, and Erbb2—the latter related to cell motility and Ptk6 activation), Tgf-β receptor (involved in epithelial to mesenchymal transition), and Gpcr (chemokine receptors bind chemokines).

This Reactome is available in the supplementary material (.json data). 

## 3. Discussion

Cell migration is a necessary event for wound healing and essential during re-epithelization [25], so the previous results obtained after treatment with 50 µg/mL of dermatan sulfate indicated an increase in epithelial fibroblasts at the site of the experimental lesion in vitro, after 24 h incubation, a fact that ensured an adequate supply of cells to migrate and cover the lesion surface [21].

In the skin of animals treated with DS (Figure 6A), the presence of reticular collagen fibers (stained blue in Masson’s trichrome stain) signals the formation of mature fibers. Furthermore, the presence of a hyperproliferative epidermis and the occurrence of new blood vessels throughout the dermis are characteristics of complete re-epithelialization and recovery of tissue functionality.

In physiological or pathological processes, cell growth is directly related to the blood supply, which often depends on neovascularization mediated by vascular endothelial growth factor (Vegf) [26]. However, low levels of Vegf mRNA were detected in the in vitro DS-treated cells, when compared to the control, in addition to a subtle increase in neovascularization in the treated animals (Figure 5B: D4 and D7). The occurrence of this neovascularization can be hypothetically correlated with the aberrant downregulation of Vegfa, considering the possibility of the occurrence of alternative splicing—a process that could encode pro or anti-angiogenic isoforms [27]. Another possibility for the generation of anti-angiogenic isoforms occurs through the start of the alternative translation from upstream non-AUG (CUG) codons when a C-terminally extended isoform is produced by using an alternative in-frame translation termination codon via a stop codon reading mechanism [28,29,30]. These findings suggest that treatment with 2,4-DS may have activated a regulatory mechanism via miRNA, which was previously identified as responsible for inhibiting the expression of the Vegf factor (anti-angiogenic isoform Vegfa 165b) in humans [31]. To support this hypothesis, it would be necessary to evaluate the expression levels of miR-15a-5p (e.g.), which, according to recent work, is increased during the process of blocking Vegfa and is related to decreased inflammation and fibrosis [32].

The PCR arrays technique offered an advantage in combining real-time PCR performance and the ability of microarrays to detect the expression of many genes simultaneously. This fact allowed the evaluation of 84 genes involved in various aspects of the healing path, some of which (Il6, Mapk1, Mapk3, Pten, Rac1, Rhoa, Serpine1, Tgfb1, and Tgfbr3) are also key genes for the development of the secretory phenotype associated with senescence (SASP) [33,34]. SASP is a paracrine senescence mechanism induced by various forms of stress in the microenvironments intra and extracellular, in addition to exposure to harmful external agents (ionizing radiation, wound process, etc.) or as a result of pathogenesis in many chronic diseases and conditions and with aging, which promotes an accumulation of senescent cells in various tissues [35]. Among these genes, only Mapk1 (mitogen-activated protein kinase 1, also known as Erk1) and Rhoa (Ras homolog family member A) were regulated positively by the treatment, which suggests the absence of activation of the SASP pathway, since, in this phenotype, their presence would be associated with the expression of Il6, Tgfb, and Pai-1, which did not occur. Since molecular analysis showed that there was no fluctuation in Tgfb expression between the control and the cells that received DS, these data may indicate that fibroblasts in culture did not have their biological activities interrupted in response to the stress of the healing test. Regarding its functions, Mapk1 is involved in a wide variety of cellular processes, such as proliferation, differentiation, transcription regulation, and development, while Rhoa proteins participate in the reorganization of the actin cytoskeleton and regulate cell shape, attachment, and motility [36]. 

The five constitutive genes present in the array were indicated by the supplier as endogenous controls in the murine model, as well as in the studied pathway; however, four of them suffered variation in expression when conditioned to the experimental treatment, being not chosen as housekeeping in this experiment. For replacement, the Ccl7 (mcp3) and Egfr genes were selected as references, which remained stable, in addition to the Hsp90ab1 gene, which kept its variation within the cut line CT ± 2). This fact can be explained by the nature of the genes initially selected by the supplier, which are involved in structural functions (Actb: cell motility, structure, integrity, and intercellular signaling; B2m: integrates to the cell surface and interacts with MHC class I;) or degradation of GAGs (in the case of Gusb, not identified/ expressed in both groups) [36], and therefore subject to variations due to the condition of experimental in vitro injury. For that reason, the mentioned genes were stimulated during the tissue repair process in the healing experiment (Figure 7), corroborating the increase in cell migration of murine fibroblasts treated with 2,4-DS [21]. In addition to the aspects mentioned, other studies reported that in *M. musculus*, the Actb and Gapdh genes have 69 and 197 pseudogenes throughout the genome, respectively. Since they are mostly devoid of introns and have a size similar to the original mRNA, they impair specificity during PCR reaction and, therefore, are not the most suitable as endogenous markers because there is no guarantee that they will not be transcribed and co-amplified during RT-PCR [37]. In RT-PCR experiments with human blood cells, Gapd genes were also not stable [38], whereas under experimental conditions of mechanical stress in humans, B2m had increased paracrine release in order to recruit fibroblasts via signaling by epidermal growth factor receptor [39]; both revealing a similar pattern of modulation in this study.

The process from injury until the formation of new replacement tissue is already widely known. In contrast, wounding of fetal mammalian tissue, up to the middle of the third trimester, results in scar-free healing. This phenotypic difference in comparison to adult tissue is due, in part, to reduced levels of Pdgfa, Tgf-β1, and Tgf-β2 [40]. These results are in agreement with the gene profile found for murine fibroblasts in culture submitted to the scratch assay (in both groups), which also presented low levels of Tgf family mRNA. This fact may be associated with the origin of the cells (explanted from neonate mice), because in injured adult tissues an increase in the profile of these genes has been reported [41]. Pdgfa participates in the expression of growth factors family members (Pdgf: platelet-derived growth factors; and Vegf: vascular endothelial growth factors); Tgf-β1 in cell proliferation, differentiation, and growth, and can modulate expression and activation of other growth factors; and Tgf-β2 encodes a secreted ligand of the TGF-β superfamily of proteins, would be expected [36].

Recent studies have shown that wounding in *Mus* elicits a strong inflammatory response [42], as demonstrated by the mRNA levels evaluated in this study that were increased for the genes of Cd40lg (regulates B cell function), Cxcl11 (has effects on endothelial cells involved in angiogenesis or angiostasis, as well as chemotaxis of T cells), Ifng (acts during embryonic development) and Il1b (produced by activated macrophages, mediates the inflammatory response and regulates cell proliferation and differentiation). In contrast, the mRNA levels of the Il4 (mediates the host’s inflammatory responses), Cxcl3 (plays role in inflammation), and Ccl12 (involved in allergic inflammation and the host response to pathogens) genes were decreased by treatment, while the expressions of Cxcl5 (promotes angiogenesis and remodels connective tissues), Il3 (acts as cell growth promoting), Il6 (implicated in a wide variety of inflammation-associated disease states) and Il10 (has pleiotropic effects in immunoregulation and inflammation) have not been changed [gene functions provided by RefSeq] [36]. These data suggest that the treatment increased cytokine and chemokine expression related to cellular development processes, along with an ECM production profile, which represents a favorable microenvironment for tissue repair.

The analysis of the Reactome indicated that the cells treated with 2,4-DS responded to the healing event by activating the main pathways of this process, already described in Figure 1, prioritizing those that correspond to phases “b” and “c” (hemostasis), “d” (inflammation), and “e” and “f” (extracellular matrix synthesis). However, it is worth mentioning that although there is an increase in the expression of genes that regulate the hemostasis pathways, it has already been identified that 2,4-DS does not activate platelet aggregation both in vitro and in vivo, and on the other hand, is also free of hemorrhagic reaction [22], which indicates an advantage of a potential therapy with this compound.

These data identified a candidate compound for additional in vivo experiments in order to elucidate the mechanisms of 2,4-DS activity in wound healing events. The initial tests presented in this work on a small number of animals under an experimental wound protocol showed that the healing profile observed in in vivo models has progressed satisfactorily in the presence of 2,4-DS, supplanting the action of saline solution in the control group. Despite this, the angiogenic potential demonstrated by DS in the treatment of wounds leads to a good prognosis of the healing process, since neovascularization indicates the conservation of tissue function [43].

Notwithstanding the high degree of identity between the genes analyzed in the RT-PCR array and the corresponding orthologs in humans, it must be emphasized that the mechanism for repairing mice skin is not completely equal to that of humans. In addition, a study that compared the skin of humans and mice, by gene array, revealed 30.2% identity. Taking these particularities into account, and considering that between the two species conserved genes have already been identified, which encode structural proteins and molecules related to cell proliferation [44], the murine model still represents an important tool in basic research both for economic aspects and for reproducibility and should not be disregarded [45].

The results obtained with 2,4-DS in wound healing indicate promising bioactive characteristics. Therefore, it is necessary to invest in the presentation of this potential treatment, as well as in improving the process of obtaining it to increase the yield of the compound.

## 4. Materials and Methods

### 4.1. Polysaccharides Obtaining

Ascidians of the specie *Styela plicata* were collected in Rio de Janeiro, Brazil. The viscera were detached from the tunic through a transverse cut made in the body of each specimen and delipidated by solubilization in acetone PA, kept in a refrigerator at 4 °C for a period of 24 h, after which the supernatant was discarded. This process was performed three times. After delipidation, the material was dried for 4 h in a drying chamber at 60 °C, sprayed, and weighed, obtaining the ketone powder. The dry viscera powder (20 g) from each specimen underwent proteolytic digestion buffer (100 mM sodium acetate, pH 5.5, containing 5 mM EDTA and 5 mM cysteine) with 10% papain, in the ratio of 20 mL/g of material to be digested, for 18 h at 60 °C in a water bath. The digestion product was centrifuged at 3700 rpm for 7 min at 20 °C, and the supernatant was collected. The precipitated, containing tissue debris, was digested again under the same conditions described above. This proteolytic digestion process was repeated a total of three times. The sulfated polysaccharides present in the supernatant were precipitated with 0.5% cetylpyridinium chloride (CPC) and absolute ethanol. After 24 h at room temperature, the mixture was centrifuged at 3700 rpm for 7 min at 20 °C. The supernatant was discarded and the precipitate was washed with approximately 10 mL of distilled water. The material was solubilized in a 2:15 NaCl: ethanol solution in the ratio 85:15 (*v*/*v*), to dissociate the CPC from the polysaccharides. Then, ethanol was added until a concentration of 50% was reached in the solution, allowing the precipitation of all polysaccharides that were collected by centrifugation at 5000 rpm for 10 min. The supernatant was discarded and the precipitate lyophilized, thus obtaining the total polysaccharides extracted from the viscera of the ascidians, as described in the protocol for sea cucumber polysaccharides [46].

### 4.2. Identification, Purification, and Isolation of Dermatan Sulfate

The extracted GAGs were identified by electrophoresis in 0.5% agarose gel and 1.3-diaminopropane/50 mM acetic acid buffer, pH 9 (100 V for 1 h). A standard aliquot of chondroitin, dermatan, and heparan sulfate was used. At the end of the electrophoresis, the polysaccharides were fixed on the gel by incubation in 0.1% cetyltrimethylammonium bromide (cetavlon). After 3 h, the gel was dried under heat using a light source and the sulfated polysaccharides were stained with 0.1% toluidine blue and acetic acid:ethanol:water (0.1:5:5, *v*/*v*). The GAGs of the total polysaccharides obtained and identified in the previous step were separated by selective precipitation induced by reducing the polarity of the solvent with ethanol. The lyophilized material was resuspended in 2% sodium chloride, at a concentration of 10 mg/mL, adding absolute ethanol to this solution until reaching the final concentration of 25%. After the period of 12 h at 4 °C, the mixture was centrifuged at 3700 rpm for 7 min at 20 °C, its supernatant was collected, and the precipitate was reserved (this being GAG fraction 1). The same volume of absolute ethanol as in the previous step was added to the collected supernatant, obtaining a concentration of approximately 40% ethanol in the mixture. Again, the mixture was left to stand for a period of 12 h at 4 °C, at the end of which it was centrifuged in the same parameters when fraction 2 of the polysaccharides was collected. The process was repeated once again, leading to a final concentration of 50% ethanol, when fraction 3 was collected. The fractions were dialyzed in distilled water and lyophilized. The GAGs present in the three fractions were identified by agarose gel electrophoresis [47].

In parallel, the polysaccharides were separated from the mixture directly by chromatographic analysis by ion exchange (Jasco) in a Sepharose QXL ion exchange column (GE Biosciences, USA), conditioned in 0.02 M Tris-HCl buffer, pH 8, containing 5 mM EDTA. The GAGs were eluted with a linear gradient, whose mobile phase consisted of sodium chloride solution (0.5–1.3 M), a flow rate of 1 mL/min, and a column temperature of 40 °C. Fractions of 0.5 mL were collected and analyzed for their metachromatic property (absorbance at 525 nm) in a Shimadzu spectrophotometer, using 1,9-methylene blue (DMB). The fractions corresponding to each peak were grouped, dialyzed in distilled water, and lyophilized. Subsequently, the identity of each GAG was confirmed by agarose gel electrophoresis. 

The polysaccharide extracts (approximately 15 µg) were incubated with 0.01 U/mg (of GAG) of chondroitinase AC or ABC in 10 µL of 50 mM ethylenediamine buffer, 82 mM acetic acid, pH 7.0, at 37 °C, for 18 h, and every 5 h, an aliquot of 0.01 U/mg was added to the incubation solution. At the end of the digestion process, the material was lyophilized and resuspended in ultra-pure water at a concentration of 10 mg/mL. The extent of depolymerization and enzymatic degradation were evaluated by agarose gel electrophoresis, applying and comparing the intact and degraded material. The described process was followed by the identification and characterization of the disaccharide units by ion exchange liquid chromatography. The formed disaccharides were recovered by filtration gel chromatography using a Superdex 75 column, connected to the Jasco HPLC. Elution was performed with 20% aqueous acetonitrile solution, pH 3.5, at a flow rate of 0.5 mL/min. The analytes were monitored in a 232 nm photodiode array detector. Fractions of 2 mL were collected, and those containing the disaccharides were lyophilized and resuspended in ultra-pure water at a concentration of 10 mg/mL and analyzed by ion exchange chromatography using a Spherisorb-SAX column, connected to the Jasco HPLC system. Elution was performed with a linear gradient (0 to 1 M) of NaCl solution, at a flow rate of 0.5 mL/min and the absorbance was monitored at 232 nm. To identify the disaccharides, the analyzed material was compared, regarding the column retention time, with the following disaccharide patterns: α-ΔHexUA-GalNAc; α-ΔHexUA(2S)-GalNAc; α-ΔHexUA-GalNAc(6S); α-ΔHexUAGalNAc(4S); α-ΔHexUA(2S)-GalNAc(6S); α-ΔHexUA-GalNAc(4,6S); α-ΔHexUA(2S)-GalNAc(4S); and α-ΔHexUA(2S)-GalNAc(4,6S).

### 4.3. In Vivo Healing Model

Wound healing can be assessed both by clinically observed changes and histological parameters. Thus, it was decided to use the excisional wound-splinting model in mice [6,48,49]. Male C57Bl/6 mice aged 03 months and average weight of 25 g were used for the experimental study, housed on a 12 h light/dark cycle in animal housing facilities and grouped in micro-isolators, receiving pelleted irradiated ration, and filtered water ad libitum throughout the experimental period.

To perform the aseptic surgical wound, the animals were anesthetized with 10% ketamine (100 mg/kg) and 10% morphine (5 mg/kg), shaved in the dorsal region with depilatory cream (Veet^®^). Subsequently, a silicone ring (14 mm of external diameter, 10 mm of internal diameter, and 0.5 mm of thickness) (Silicone WoundSplint, Grace Bio-Labs^®^) was subcutaneously implanted in the dorsal region of the mouse. The ring was fixed with 8 discontinuous points, using 5.0 non-absorbable nylon thread for suturing. After fixation, the skin contained in the inner diameter of the ring was removed with the help of Iris scissors. The images of the wounds were recorded using a digital photographic camera (Nikon DSLR 610), fixed on a tripod with a standard distance of 30 cm from the wound maintained for all animals.

The animals were separated into 2 groups (N = 4/group): the control group, which was treated only with saline solution (NaCl: 0.9%), and the test group, which received topical treatment with 0.25% dermatan in saline suspension. In addition, skin biopsies from mice that did not participate in the experiments served as a reference for histological comparison. After receiving 20 μL of the corresponding treatment solution, a dressing was applied with a hypoallergenic absorbent adhesive 10 mm in diameter (Stopper, Proinlab^®^), wrapping it with a bandage based on a transparent polyurethane film and a protective layer of poly-terephthalate ethylene (Vitalderm^®^), covering the entire back of the animal. The animals were kept individually in microisolators, thus avoiding the possible removal of dressings by other mice.

On days 2, 4, 7, 11, 14, and 17 after the procedure, the individuals were sedated with isoflurane in order to perform dressing change with reapplication of treatment and photographic record of the wounds. The macroscopic evaluation of the wounds was performed on days 4, 7, and 17 post-surgery, corresponding to the inflammatory, fibroblast, and remodeling stages, respectively. The collected parameters, such as the presence of: crust, fibrin, granulation tissue (location and quality), and healing; gave rise to an ordinal measurement scale, with scores ranging from zero (poor healing) to 12 (clinically healed wound) inspected by an independent evaluator (single-blind) (Table 1).

The surgical wound area was based on the epithelialized borders and was measured on days 0, 4, 7, 11, 14, and 17, with the aid of the ImageJ^®^ software. On the 17th day, after the photographic record, the animals were euthanized by administering isoflurane without oxygen followed by cervical dislocation, and then, tissue samples were collected corresponding to the region of the surgical wound with a 0.5 cm skin margin around the lesion. The material was fixed in 10% formaldehyde and included in paraffin for later obtaining of histological slides. Histological sections (5 μm thick) were stained with hematoxylin–eosin (H&E) and Masson’s trichrome. The images of the slides were captured in the equipment Scanner 3D Histech and visualized with Pannoramic Viewer Software. The histological parameters evaluated were selected based on those most addressed in similar studies [50], generating the histological evaluation (Table 2). The result of the average score between the five selected parameters classifies the wound as poorly healed (value 3), in the healing process (value 2), and healed/re-epithelized wound (value 1).

**Table 2:** Histological parameters for skin wound healing assessment.

Parameters	Classification	Score
Amount of granulation tissue	Profound	1
	Moderate	2
	Absent/mild	3
Amount of collagen	Large amount	1
	Moderate	2
	Absent/ mild	3
Collagen pattern	Reticular	1
	Mixed	2
	Fascicle	3
New vessels	Marked	1
	Moderate	2
	Absent/mild	3
Re-epithelization	Total	1
	Partial	2
	Absent	3

The result of the average score between the five selected parameters classifies the wound as poorly healed (value 3), in the healing process (value 2), and healed/re-epithelized wound (value 1).

### 4.4. The Wound Healing Pathway-Focused Gene Expression Using Real-Time PCR

Primary cultures of fibroblasts from C57BL/6 neonate mice were extracted from the dermis by explant technique, as described by Carrel and Burrows [51]. Initially, fragments of mice skin were removed by biopsy and reserved in buffer saline phosphate (PBS) containing 10,000 U/mL penicillin antibiotic solution, streptomycin 10,000 µg/mL (1%), and antimycotics (amphotericin B—1 µg/mL) (Sigma Aldrich); and kept at 4 °C for 30 min. Then, they were washed in PBS and sectioned into fragments of approximately 1 mm². The dermis fragments were adhered to the surfaces of vials for the culture of cells with 25 cm² areas, and grown in Dulbecco’s Modified Eagle Medium (DMEM), containing 10% fetal bovine serum, and 1% solution antibiotic antimycotic (Sigma Aldrich). The culture medium was changed every three days and fibroblast cultures were kept in incubator in an atmosphere of 5% CO_2_ until the experimental tests are carried out.

Fibroblast cultures between the 3rd and 5th passage were plated in 35 mm plates with a density of 2 × 10^5^ cells per plate in DMEM. After the cells reached about 80% of adhesion and confluence, which occurred 24 h later, perpendicular scratches were performed in the monolayer with the aid of a 200 µL tip (scratch assay), removing the loose cells by washing with PBS. Simple DMEM (experimental control) or DS (2,4-DS from *S. plicata*) medium was added to each plate, at a concentration of 50 µg/mL. Throughout the experiments, mitomycin C (Sigma) (50 µg/mL) inhibited cell proliferation during the incubation time. The cells were incubated for 48 h and then, the fibroblasts 2,4-DS-treated and the control group had their total RNA extracted with RNEasy Plus Mini Kit (cat. nº. 74104, Qiagen). In this step, about 1 × 10^5^ cells were resuspended in DMEM culture medium and subjected to centrifugation at 14,000 rpm for 5 min, according to the manufacturer’s instructions. The purification yield was measured by spectrophotometry using the Qiaxpert nucleic acid quantifier (Qiagen), where the RNA concentrations obtained were evaluated and the degree of purity was estimated, considering the absorbance ratios measured at 260 nm and 280 nm, which should be equal to or greater than 1.75. For the following tests, samples considered to be pure were selected, which presented an optical density between 1.8 to 2.0. An additional step was performed with DNAse treatment to eliminate the remaining gDNA (starting from 583.2 ng nucleic acid in a final volume of 10 µL, for all samples). This gDNA elimination mix was incubated for 5 min at 42 °C and subsequently subjected to an ice bath for 1 min. To obtain the complementary DNA (cDNA), the samples were incubated with the RT^2^ First Strand Kit (cat. nº. 330401, Qiagen). An optional quality control step was performed before the gene expression experiment. The RT^2^ RNA QC PCR Array (cat. nº. 330291, Qiagen) evaluates high and low housekeeping gene expression levels, contamination with DNA (genomic and general), and reverse transcription/ polymerase chain reaction efficiency. 

The relative quantification experiment was also performed in biological triplicates, with a randomized design (test group: treated with 50 µg/mL of 2,4-DS and control group: supplemented with DMEM), following the criteria recommended by the MIQE guidelines [52]. For this assay, the cDNA was used on the real-time mouse wound healing RT² Profiler PCR array (cat. nº PAMM-121Z, Qiagen) in combination with RT² SYBR Green qPCR Mastermix (cat. nº 330529, Qiagen), which allows the evaluation of the expression of 84 genes participants of the wound healing response, as described in manufacturer’s datasheet. The kit includes the Hot Start DNA Taq polymerase, in addition to the SYBR green and ROX dyes (passive reference). To normalize the target genes, five housekeeping or reference genes (endogenous controls) were evaluated, constituting this model (Actb, B2m, Gapdh, Gusb, and Hsp90ab1). In addition, a gDNA contamination control, three reverse transcription controls (first strand synthesis RTC), and three positive PCR controls (PPC) of the polymerase chain reaction efficiency were used (this last control consists of a pre-dispensed artificial DNA sequence). The remaining four positions of the 100-well ring were allocated to the no-template control (NTC). Reverse transcription and amplification were performed on the Rotor-gene Q 5PLEX thermocycler (Qiagen GmbH), in a final volume of 20 µL per reaction, using the following thermal profile: step 1—95 °C for 600 s; step 2—95 °C for 15 s; step 3—60 °C for 30 s (acquiring); step 4—go to step 2 loop (repeat 44 times); step 5—gradient temperature: 99 °C to 50 °C for 245 s, ramp: 0.2 °C/s. Was realized a dissociation (melting) curve analysis to verify PCR specificity with the following program: 95 °C, 1 min; 65 °C, 2 min (optics off); 65 °C to 95 °C to 2 °C/min (optics on); to generate a first derived dissociation curve for each well using the real-time cycler software. A single peak was expected in each reaction at temperatures above 80 °C.

To determine the cycle threshold (CT), the following parameters were used for both groups in the Rotor-Gene Q Series Software version 2.3.1 (build 49): ignore the first three cycles; threshold 0.02024; eliminate cycles before 4.95. CT values were exported to the data analysis web portal at http://www.qiagen.com/geneglobe. Samples were assigned to controls and test groups. The selected CT cut-off was 36. After calculating the arithmetic mean of the CTs for each gene, the CT values of the test group were normalized based on an automatic selection of the panel of reference genes, considering those present at baseline concentrations both in the control and test group. The data analysis web portal calculated fold change/regulation using the ΔΔCT method, in which ΔCT is calculated between the gene of interest and an average of reference genes (HKG, housekeeping), followed by Δ-ΔCT calculations [ΔCT (test group) − ΔCT (control group)]. Fold change is then calculated using the 2^(−ΔΔCT)^ formula, as described by Livak and Schimittgen [53]. The *p*-values are calculated based on a Student’s *t*-test of the replicate 2^(−ΔCT)^ values for each gene in the control group and treatment groups [54].

## Figures and Tables

**Table 1:** Scale for the ordinal measurements of wound parameters.

Wound Parameters	Absence	Presence	Score
Crust	1	0	0–1
Fibrin	1	0	0–1
Granulation	0	1	0–1
Localization	N/A	Whole wound surface= 3 Border = 2 Middle = 1	1 to 3
Quality	N/A	Bright red color = 2 Pale = 1	1 to 2
Healed	0	9	Ausence = 0 Presence = 9
Closed wound *	0	10	Ausence = 0 Presence = 10

* Remodeled skin, without crust, and with the presence of hair.

## Data Availability

The array dataset generated can be found in the NCBI’s Gene Expression Omnibus data repository, accession number GSE160142 [https://www.ncbi.nlm.nih.gov/geo/query/acc.cgi?acc=GSE160142].

## Supplementary Materials

The following supporting information can be downloaded at: https://www.mdpi.com/article/10.3390/md20110676/s1, Table S1: arithmetic mean and standard deviation of the healing area; Table S2: assay of quality control of the RNA; Figure S1: quality control of mRNA expression levels of housekeeping genes; Table S3: up-down regulation of genes on DS-treated group (comparing to the control group); Reactome (.json data).
