# Supplementary material for: Effects of Dermatan Sulfate from Marine Invertebrate Styela plicata in the Wound Healing Pathway: A Natural Resource Applied to Regenerative Therapy
Source: Mar Drugs. 2022 Oct 28;20(11):676. doi: 10.3390/md20110676 (PMC9693086; doi:10.3390/md20110676)
Supplement: Supplementary file 1 [file marinedrugs-20-00676-s001.zip › marinedrugs-1921068-supplementary.pdf]

Table S1: Arithmetic mean and standard deviation of the healing area.
Table S2: Assay of quality control of the RNA
Figure S1: Quality control of mRNA expression levels of housekeeping genes.
Table S3: Up-down regulation of genes on DS-treated group (comparing to control group)

Table S1: Arithmetic mean and standard deviation of the healing area

Days after wound	Control group			Test group (DS treatment)		
	arithmetic average	Standard deviation	N	arithmetic average	Standard deviation	N
0	1045,75	85,49	4	1022,67	147,31	3
2	769,75	104,47	4	927,25	45,13	4
4	713,5	113,11	4	812,25	59,02	4
7	584,5	155,59	4	658	124,97	4
9	375	72,95	4	403,75	40,09	4
11	144,75	95,17	4	230,5	121,71	4
14	62,5	86,26	4	132	90,26	4
17	17	20,96	4	18	36	4

N = number of mice per group.

Table S2: Assay of quality control of the RNA

	Sample Name	S1	S2	S3	S10	S11	S12
Row	Internal controls	Ave Ct					
A	ACTB*	30,48	20,38	22,74	17,29	20,52	17,06
B	HPRT1*	17,02	17,49	19,46	17,04	18,31	16,88
C	RTC	21,59	35	35	20,11	21,35	22,37
D	PPC	18,38	18,34	18,23	17,3	17,96	17,87
E	GDC	35	35	35	35	35	35
F	NRT	35	31,22	35	35	35	32,78
G	PPC - H2O	19	18,63	18,14	35	35	35
H	NTC	35	35	35	35	35	35
	Reverse Trascription Control (RTC): Are RNA sample impurities affecting reverse transcription?						
	CtC - CtG	2,59	16,37	16,86	-14,89	-13,65	-12,63
	Cut-off 5	NO	Perhaps	Perhaps	NO	NO	NO
	Positive PCR Control (PPC): Are RNA sample impurities affecting PCR?						
	CtD - CtG	-0,62	-0,29	0,09	-17,7	-17,04	-17,13
	Cut-off 3	NO	NO	NO	Perhaps	Perhaps	Perhaps
	No Reverse Transcription (NRT) control: Is genomic DNA contamination detectable?						
	CtF	35	31,22	35	35	35	32,78
	Cut-off 35	NO	YES	NO	NO	NO	YES
	Genomic DNA Contamination (GDC) control: Will it affect the results?						
	CtE	35	35	35	35	35	35
	Cut-off 35	NO	NO	NO	NO	NO	NO
	No Template Control (NTC): Is the PCR system clean?						
	CtH	35	35	35	35	35	35
	Cut-off 34	YES	YES	YES	YES	YES	YES

CT: Cycle Threshould

S1-S3 and S10-S12: RNA samples (control and treatment groups)

*Housekeeping genes

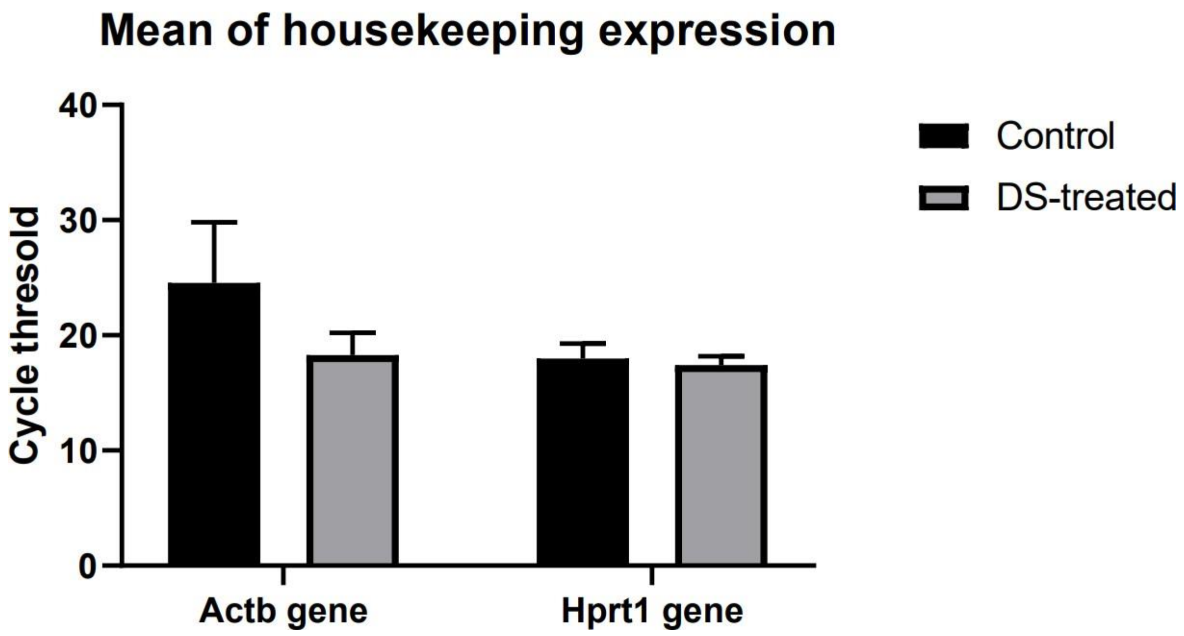

Figure S1: Quality control of mRNA expression levels of housekeeping genes. Analysis of two constitutive genes actin beta and hypoxanthine phosphoribosyltransferase 1 (Actb and Hprt1, respectively) were detected, which demonstrated stable baseline concentration of Hprt1 and non-statistical variation of the Actbgene.

Table S3: Up-down regulation ofgenes on DS-treated group (comparing to control group)

Gene	Fold Regulation	p-value
Acta2	1.01	0.874869
Actc1	1.01	0.874869
Angpt1	**-1153.08**	**0.018693**
Ccl12	**-122120.35**	**0.013677**
Ccl7	-1.23	0.408155
Cd40lg	**17.29**	**0.005399**
Cdh1	1.01	0.874869
Col14a1	1.81	**0.047092**
Col1a1	**2584.00**	**0.007833**
Col1a2	**7924.23**	**0.001016**
Col3a1	**69.33**	**0.000039**
Col4a1	**5.32**	**0.032162**
Col4a3	**-1535.63**	**0.004485**
Col5a1	1.01	0.874869
Col5a2	**4.59**	**0.004717**
Col5a3	**4.26**	**0.013494**
Csf2	**8.12**	**0.000069**

Csf3	**35.89**	**0.001219**
Ctgf	**91.06**	**0.033101**
Ctnnb1	**26.58**	**0.009025**
Ctsg	**2.35**	0.099407
Ctsk	**4.98**	**0.004561**
Ctsl	1.01	0.874869
Cxcl1	**119119.98**	**0.010777**
Cxcl11	**42.58**	**0.021374**
Cxcl3	**-6002.21**	**0.015707**
Cxcl5	1.01	0.874869
Egf	**-49.68**	0.137766
Egfr	1.64	**0.022645**
F13a1	**1099.06**	**0.035764**
F3	**705.28**	**0.024809**
Fga	**11.57**	**0.035049**
Fgf10	**15.99**	0.113947
Fgf2	**229.45**	**0.013049**
Fgf7	**3393.89**	**0.006725**
Hbegf	**43.47**	0.063086
Hgf	**-38.26**	**0.000513**
Ifng	**148.61**	0.108057
Igf1	**14.74**	**0.026152**
Il10	1.01	0.874869
Il1b	**1590.64**	**0.015092**
Il2	1.01	0.874869
Il4	**-4.30**	**0.000508**
Il6	1.01	0.874869
Il6st	**91.48**	**0.003735**
Itga1	**671.88**	**0.016488**
Itga2	**115328.47**	**0.028990**
Itga3	1.01	0.874869
Itga4	**-4.19**	**0.001601**
Itga5	**-8.46**	**0.001387**
Itga6	1.01	0.874869
Itgav	**3.96**	**0.006900**
Itgb1	1.50	0.207414
Itgb3	1.01	0.874869
Itgb5	1.01	0.874869
Itgb6	**77555326.11**	**0.000338**
Mapk1	**40705559.86**	**0.001989**
Mapk3	**46299.93**	0.133961
Mif	**99017.04**	0.172904
Mmp1a	**-6507.75**	0.053707
Mmp2	**80.19**	0.070737
Mmp7	1.43	0.231791
Mmp9	**172.69**	0.058591

Pdgfa	1.01	0.874869
Plat	**3.16**	**0.023533**
Plau	1.25	0.167182
Plaur	**485.07**	0.075561
Plg	**12.60**	**0.014003**
Pten	**139.95**	0.052366
Ptgs2	**19154.41**	**0.023692**
Rac1	**65.14**	0.207252
Rhoa	**1346.86**	**0.041658**
Serpine1	1.01	0.874869
Stat3	**91114.26**	**0.048767**
Tagln	**9087104.21**	**0.046402**
Tgfa	**165.27**	**0.014793**
Tgfb1	1.01	0.874869
Tgfbr3	1.01	0.874869
Timp1	1.01	0.874869
Tnf	1.01	0.874869
Vegfa	**-3527.94**	0.171114
Vtn	**-3471.34**	**0.002632**
Wisp1	1.01	0.874869
Wnt5a	**-43.35**	0.165277
Actb	**14727976.14**	**0.021116**
B2m	**27276.85**	0.128710
Gapdh	1.01	0.874869
Gusb	1.01	0.874869
Hsp90ab1	-1.26	0.284365

Bold genes: threshold of fold regulation and p-valuesare 2 and < 0.05, respectively.
